# Foreign Body Ingestion in Children: From a Harmless Incident to a Life-Threatening Emergency

**DOI:** 10.3390/life16071172

**Published:** 2026-07-16

**Authors:** Maria-Delia Mihailov, Ioana-Cristina Olariu, Vlad Laurențiu David, Gabriela Simona Doros

**Affiliations:** 1 Faculty of Medicine, Victor Babes University of Medicine and Pharmacy, 300011 Timisoara, Romania; olariu.cristina@umft.ro (I.-C.O.); david.vlad@umft.ro (V.L.D.); doros.gabriela@umft.ro (G.S.D.); 2 Pediatric Intensive Care Unit, Louis Turcanu Emergency Children’s Hospital, 300011 Timisoara, Romania; 3 IIIrd Pediatric Clinic, Louis Turcanu Emergency Children’s Hospital, 300011 Timisoara, Romania; 4 Department of Surgery, Louis Turcanu Emergency Children’s Hospital, 300011 Timisoara, Romania

**Keywords:** foreign body, ingestion, pediatric emergency, children, imaging, button battery, magnets, endoscopy, foreign body management, complications

## Abstract

Accidental foreign body ingestion is a common and often harmless event in childhood, especially among very young children, who naturally explore their surroundings by putting objects in their mouths. However, certain objects (button batteries, magnets, sharp objects), once ingested, pose a risk of complications, sometimes potentially life-threatening. Awareness of this risk is particularly important, as their removal must be performed as soon as possible in a specialized center. This narrative review aims to provide an overview of the most recent data on the epidemiology, clinical and imaging diagnosis, and particularly the therapeutic approach to foreign body ingestion in children. A comprehensive review of the literature was conducted to identify the most relevant evidence in this field, aiming to analyze situations in which foreign body ingestion constitutes an emergency and to present the appropriate diagnostic and therapeutic approach in specific cases. The degree of urgency is determined by the type of ingested object, its location in the digestive tract, and symptoms suggestive of complications; these situations are analyzed and described in detail to serve as a useful guide in medical practice. Preventive measures are important in avoiding these life-threatening situations, and therefore, parents and caregivers must be informed and take steps to prevent children from accessing dangerous objects.

## 1. Introduction

Accidental ingestion of foreign bodies (FBs) is a common occurrence in childhood; in young children, introducing objects into their mouths is a normal and necessary phase of exploration and development [1]. In older children, it predominantly occurs in those with psychological or neuropsychological disorders [2].

## 2. Study Design

A narrative review of the literature was conducted using the PubMed/MEDLINE database. Filters were applied to identify studies on FB ingestion in children published in the last 10 years, involving human subjects aged 0–18 years, available in English. Eligible articles included clinical trials, observational studies, reviews, systematic reviews, meta-analyses, and clinical practice guidelines. After a careful examination and a full-text evaluation, we selected 37 articles based on their relevance, credibility, and significance to the topic.

## 3. Epidemiology

In most cases, ingested FBs are evacuated through the stool within a few days without complications; endoscopic removal is necessary in only 10–20% of cases, and surgical intervention in less than 1% of patients. The majority of ingestions are accidental, particularly in children younger than 5 years old, with the highest incidence reported in those aged 2–3 years [3,4].

In recent years, the incidence of accidental ingestion of button batteries and magnets has increased significantly, due to their growing use and their presence in children’s toys [5,6].

## 4. Pathophysiology

Complications are more likely in children with pre-existing gastrointestinal (GI) tract disorders, such as esophageal or duodenal stenosis, previous surgeries, or abnormal GI motility. Still, they are also reported in children with no known risk factors, some of them having previously unidentified anatomical or functional abnormalities [7]. Most FBs can become lodged in the esophagus at physiological strictures, such as the thoracic inlet, the aortic arch in the middle esophagus, and the gastroesophageal junction, leading to varying degrees of obstruction.

Button batteries, particularly the button type, can react with saliva and moist esophageal mucosa, generating a local current that triggers electrical hydrolysis, releasing hydroxyl radicals at their negative pole. This reaction can cause severe damage through corrosion, resulting in burns that can occur within just one hour, followed by severe coagulative necrosis. Voltage and, importantly, the duration of contact are key factors in determining the severity of the injury.

Lithium batteries, in particular, can generate higher voltages and currents, leading to increased hydroxide accumulation. Other mechanisms by which complications arise include local pressure necrosis, corrosive injuries caused by battery leakage, and heavy metal poisoning [8,9].

Neodymium magnets are 20–30 times stronger than standard magnets. Even if swallowed separately and passing through the GI tract individually, they strongly attract each other across the intestinal walls, causing bowel obstruction, perforation, and fistula due to pressure necrosis [10].

A particularly serious situation arises when a magnet and a button battery are swallowed together, as the combination of injuries from local current flow and erosion from the strong magnetic attraction can lead to severe complications.

Superabsorbent polymers, derived from hydrophilic acrylic acid polymers, are those water-filled bead toys that can increase their initial volume by up to 60 times when ingested, expanding considerably in wet conditions, as well as diapers and feminine hygiene products. Their ingestion poses significant risks, such as intestinal obstruction, perforations, and even death, usually requiring surgical intervention for their removal [11].

Sharp objects can become lodged at the base of the tongue, in the vallecula, in the tonsillar fossa, in the pyriform sinuses, or can perforate the pharyngeal wall and penetrate deep structures [12]. In fact, they can cause perforations at any level of the digestive tract.

A trichobezoar results from chronic hair ingestion (one’s own, from dolls, or from pets), a behavior called trichotillomania. This can fill the stomach and extend into the duodenum or even the colon. Inspired by the Brothers Grimm fairy tale, the extension of the bezoar into the small intestine is called “Rapunzel syndrome” [13].

## 5. Evaluation

### 5.1. Clinical Evaluation

In all cases, the clinical examination must be preceded by a detailed medical history. However, the caregivers may not have noticed the ingestion, and therefore, clinical suspicion is extremely important.

Most FBs pass through the GI tract without causing any complaints. When symptoms do occur, they are mild and nonspecific: abdominal pain, nausea, vomiting, or a sensation of abdominal fullness. Severe symptoms arise from complications and depend on the type of FB, the time since ingestion (e.g., for batteries), the location where they are lodged, and any preexisting conditions of the patient. In all children, after a detailed oropharyngeal examination, it is also important to assess the airway to exclude a proximal airway obstruction.

An object lodged in the esophagus can cause complications manifested by chest pain, a FB sensation, coughing, dyspnea, odynophagia, dysphagia, and vomiting. In severe cases, hematemesis or signs of mediastinitis may occur. Complete obstruction of the esophagus can lead to excessive salivation, which can indirectly compromise the airways due to the accumulation of secretions and the risk of aspiration. Complications occurring in the stomach or gut result in abdominal pain, vomiting, or GI bleeding [14].

Trichobezoar symptoms include abdominal pain, growth disorders or weight loss, anorexia, vomiting, anemia, GI hemorrhage, or symptoms of ileus or perforation [15].

It is important to note that FB ingestion should be considered in any child admitted to the hospital with digestive or respiratory symptoms that do not respond to treatment.

### 5.2. Imaging Evaluation

Plain radiographs of the neck, thorax, and entire abdomen are the first-line choice for identifying the type, size, and location of ingested objects, especially the radiopaque ones (batteries, magnets, coins, etc.), though they are less effective for radiolucent materials such as food fragments, fish bones, or glass shards.

Radiographic investigation (X-ray) is also indicated for detecting complications such as esophageal perforation (pneumomediastinum) and gastric or intestinal perforation (pneumoperitoneum).

An important point is that button batteries can be confused with coins on a simple X-ray, leading to management errors with potentially catastrophic consequences. The batteries appear on chest X-rays as a “double contour” or “halo” sign in the anteroposterior projection due to their architecture with an upper part (positive pole) and a lower part (negative pole), creating two concentric outlines.

Multiple neodymium magnets can appear as a single magnet on X-ray; in these cases, lateral X-rays should be taken to confirm their number. Serial X-rays allow monitoring of the displacement and elimination of radiopaque bodies. Furthermore, lateral neck X-rays play a crucial role in revealing edema or inflammation, potentially caused by FB impaction and perforation of the cervical esophagus [16].

Contrast radiography should not be performed when a perforation is suspected. In addition, aspiration of the contrast medium into the airways is associated with a significant risk of pulmonary edema.

Computed tomography (CT) offers greater sensitivity for identifying radiolucent objects and complications, while magnetic resonance imaging (MRI) has higher sensitivity but is not available as an emergency investigation and is mainly used for post-extraction monitoring (e.g., button batteries) [17,18].

## 6. Management

### 6.1. Indications and Timing of Endoscopy

**Figure 1:**
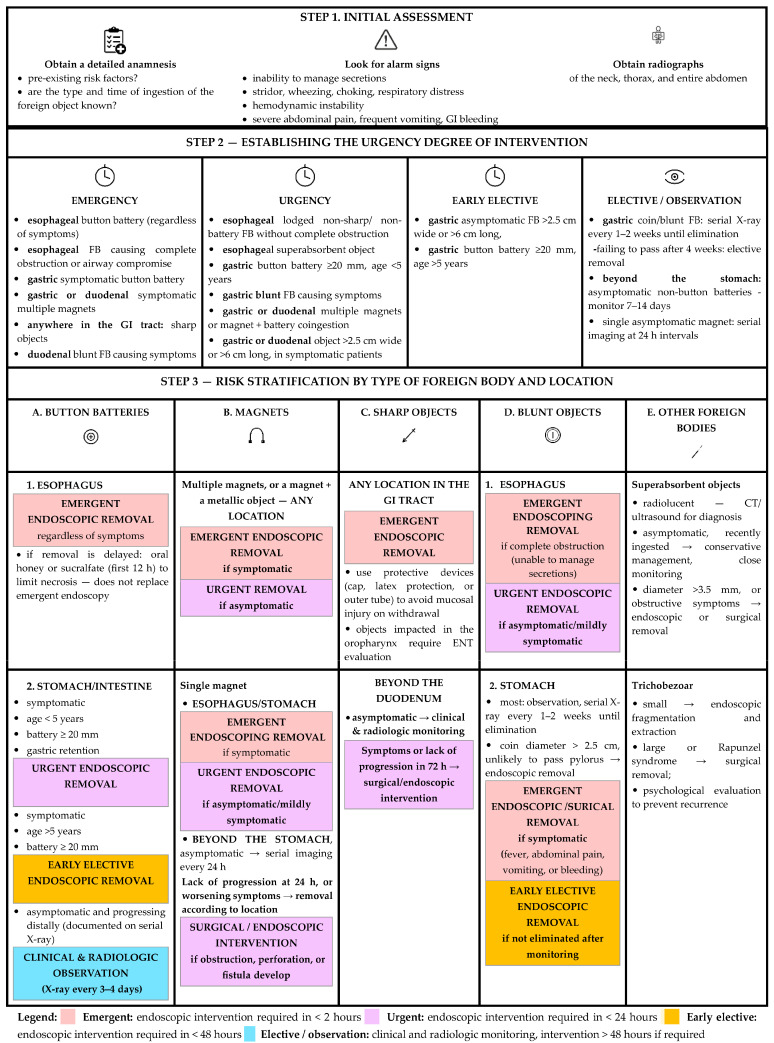
Algorithm for timing intervention in FB ingestion in children.

The management of a child who has ingested an FB begins with determining whether endoscopic removal is necessary and, if this is the case, establishing the degree of urgency. Treatment depends on the type, size, and location of the object, as well as the patient’s symptoms (Figure 1).

Conservative treatment with serial imaging and observation may be indicated for asymptomatic, previously healthy children who have ingested small, non-hazardous objects, because most often they pass spontaneously. Objects impacted in the oropharynx require ENT examination.

In healthy patients, small, inert objects near the gastroesophageal junction typically pass into the stomach within a few hours, move through the intestines, and are eliminated without complications. However, the onset of symptoms warrants urgent intervention: either endoscopic removal or surgical extraction in case of complications.

It is important to emphasize the role of the multidisciplinary team, which includes the pediatrician or emergency physician who first examined the patient, the imaging specialist, the otolaryngologist, the gastroenterologist, the anesthesiologist, and, when necessary, the surgeon.

Once the indication for endoscopy has been established, the appropriate equipment must be prepared: a standard adult flexible gastroscope with an 8.0 mm diameter is suitable for children over one year of age or weighing more than 10 kg; a smaller endoscope, <6 mm, is recommended for children weighing less than 10 kg or when esophageal stricture is suspected [19].

It should be highlighted that there is no absolute consensus among the different guidelines, but the recommendations are very similar. For example, the NASPGHAN (North American Society for Pediatric Gastroenterology, Hepatology & Nutrition) guidelines (in 2015) and the ESGE (European Society of Gastrointestinal Endoscopy) clinical guidelines (in 2016) divide procedures into three categories: major emergencies, emergencies, and non-emergencies [20,21].

Some countries have their own national guidelines. In this context, it is important to mention the guidelines of the Italian Society of Pediatric Gastroenterology, Hepatology, and Nutrition (SIGENP) and the Italian Association of Hospital Gastroenterologists and Endoscopists (AIGO), which provide clear and very useful recommendations, classifying cases into four categories of urgency: emergency (<4 h), urgency (<24 h), early elective (<48 h), and elective (>48 h) [22].

Table 1 presents the management of ingested FBs based on location, type, size, patient age, and symptoms.

### 6.2. Button Battery Ingestion

Any button battery detected in the esophagus is an emergency and requires immediate endoscopic removal. If this is not possible, oral administration of honey in the first 12 h (10 mL every ten minutes in children older than 1 year) or sucralfate (10 mL every 10 min, maximum 3 doses) is recommended to limit tissue necrosis and prevent subsequent perforation, without delaying its emergent endoscopic removal.

During endoscopy, if no local perforations are seen, the battery site should be irrigated with 50–150 mL of 0.25% acetic acid, and additional endoscopic airway evaluation should be considered in cases of extensive mucosal necrosis [23]. After endoscopic removal, an examination of the esophageal mucosa should be performed to anticipate potential complications.

All cases where the diagnosis is delayed, or there is clinical suspicion of perforation, mediastinitis, or sepsis, or where swallowing difficulties are observed, must be managed with extreme caution and monitored for several days until complications are ruled out.

Most complications occur more than 30 days after initial ingestion, so careful monitoring of these patients is important. The onset of symptoms at any time requires urgent reassessment and, in most cases, surgical intervention [24].

In cases where the battery was successfully extracted shortly after ingestion, the initial injuries may not be very severe; therefore, endoscopy should be repeated at least 48–72 h after the first evaluation [25]. Long-term follow-up after endoscopic removal depends on the presence and extent of esophageal injury.

Batteries that have passed into the stomach or beyond do not rule out esophageal injury, so this must be excluded. In these patients, a second-look examination may be considered within 2–4 days after gastric removal.

### 6.3. Magnet Ingestion

Visualization of magnets on an X-ray scan in symptomatic children requires immediate endoscopic removal if the pieces are located in the esophagus, stomach, duodenum, or colon. If endoscopic removal cannot be achieved or in case of complications, surgical removal is indicated.

In asymptomatic children, it is important to determine the number of ingested pieces. If there is a single magnet, serial imaging monitoring at 24 h intervals may be indicated to confirm its passage. If the piece does not progress in 24 h, removal should be performed depending on its location.

Asymptomatic multiple magnets can be monitored by serial X-rays every 4–6 h. The passage through the rectum should also be confirmed with a control X-ray. If no progression is observed within 6 h, urgent removal should be performed to avoid complications.

Urgent endoscopic removal of magnets ingested together with other metallic FBs is also indicated [16].

### 6.4. Sharp Object Ingestion

Sharp FBs in the gastrointestinal tract is associated with severe complications; therefore, prompt diagnosis is of paramount importance. They should be endoscopically removed without delay, using protective devices (cap, latex protection, or outer tube) to reduce the risk of retrograde mucosal injury and perforation during extraction [26].

### 6.5. Superabsorbent Object Ingestion

Superabsorbent object ingestion management is challenging because these objects are radiolucent and pass rapidly through the proximal GI tract, making CT and ultrasound important diagnostic tools. Asymptomatic patients who have recently ingested such an object may be managed conservatively under close monitoring. Endoscopy is preferred for superabsorbent objects with an initial diameter >3.5 mm [27]. Favorable outcomes have also been reported by crushing the beads during laparotomy and advancing them into the colon for enema-assisted expulsion [28].

### 6.6. Coins Ingestion

While small coins can pass through the GI tract without any problems, large coins can cause complications, similar to those caused by any other large FB.

Asymptomatic or mildly symptomatic children with an esophageal coin require endoscopic removal within 24 h, while complete esophageal obstruction requires emergency endoscopic removal.

In asymptomatic patients, gastric coins should be monitored for passage through the GI tract, with serial X-rays every 1–2 weeks, until their elimination. If they are not eliminated after this time, endoscopic removal is recommended. Coins with a diameter > 2.5 cm are unlikely to pass through the pylorus of younger children and must be removed endoscopically [29].

Symptoms like fever, abdominal pain, vomiting, or bleeding warrant prompt intervention—endoscopic retrieval or surgical removal for bowel obstructions.

### 6.7. Trichobezoar

Small trichobezoars can be extracted endoscopically after fragmentation, while larger ones often require surgical intervention [30]. Psychological evaluation and, if necessary, pediatric psychiatric therapy should always be performed to prevent recurrence [31].

### 6.8. Anesthetic Considerations

Endoscopic extraction requires general anesthesia with orotracheal intubation. In cases requiring urgent extraction, pre-anesthetic fasting (NPO or nil per os rules) is not required, as postponing the procedure can lead to serious and potentially life-threatening consequences. In these situations, airway protection is ensured by rapid sequence induction.

## 7. Complications

Although more than 80% of FBs that enter the esophagus pass spontaneously into the stomach without complications, the remainder become lodged in the esophagus. Esophageal obstruction carries the highest risk of complications (25% higher than in other parts of the digestive tract) and can be life-threatening due to its proximity to vital organs.

Patient-related risk factors that increase the risk of complications include esophageal strictures (congenital or acquired), prior esophageal atresia or tracheoesophageal fistula repair, eosinophilic esophagitis, motility disorders, achalasia, esophageal diverticulum, extrinsic compression by tumors, developmental, neurological, and psychiatric disorders [7].

Partial or complete obstruction of the esophagus is the most common complication, but FBs can also become lodged in any other segment of the digestive tract. Some of them may erode the GI wall, leading to perforation and subsequent migration outside the lumen. In patients with pre-existing anatomical or functional abnormalities, the risk of complications is increased [32].

Complications following battery ingestion include tracheoesophageal strictures and fistulas, mediastinitis, vocal cord paralysis, or spondylodiscitis. Button batteries larger than 20 mm in diameter can become lodged in the upper esophageal sphincter, leading to perforation and fistula formation into the major blood vessels or the trachea, which can be fatal. Perforations are typically diagnosed within 2 days (rarely within the first 12 h), but fistulas may appear up to 4 weeks after removal. Deaths can occur from fistulas in major blood vessels like the aorta, subclavian artery, or thyroid arteries, while aorto-esophageal fistulas carry a particularly high mortality rate [33].

It is important to emphasize that even after the button battery has been removed, esophageal injuries may continue to evolve for several days or weeks; necrosis of the esophagus and of the surrounding tissue is an ongoing process that can lead to fistula formation and further serious complications.

In cases of significant esophageal mucosa injury, a nasogastric tube should be inserted endoscopically to maintain the luminal patency, and the patient should not be fed orally until perforations or other complications have been ruled out.

If significant bleeding occurs during or after the impaction of a button-cell battery in the upper esophagus, fistulas to major blood vessels, such as the aorta, subclavian artery, or thyroid artery, should be considered, and immediate coordination of all available specialty departments (including cardiac and thoracic surgery, interventional radiology, and critical care) is required to establish an urgent multidisciplinary therapeutic strategy.

In all cases with severe complications, such as aorto-esophageal fistula, respiratory symptoms secondary to a tracheo-esophageal fistula, tracheal stenosis, or sepsis due to mediastinitis, complex anesthetic management is required, with invasive monitoring and subsequent follow-up in the intensive care unit [34].

According to NASPGHAN guidelines, an MRI scan has to be performed 3–5 days after the ingestion incident, followed by additional MRIs every 5–7 days, until the inflammation of the tissue surrounding the esophagus resolves, as the significant risk of fistula formation to the great blood vessels is expected to subside after 21 days. More recent data reveal that serial MRIs do not predict the development of severe complications. In one case with battery ingestion, a total of 3 MRI scans were performed on days 1, 5, and 11 after its removal, documenting a sustained improvement in mediastinal damage; nevertheless, an aorto-esophageal fistula developed after 25 days [35].

Button batteries that have passed through the esophagus should be monitored by X-ray every 3 to 4 days. If an obstruction in the small intestine is suspected, surgical intervention is indicated because, even at this stage, if they remain lodged, they can cause mucosal damage and complications.

In addition to button batteries, high-powered neodymium magnets are among the most dangerous objects that children can swallow, causing ischemia, ulceration, necrosis, perforation, fistula with consequent sepsis, obstruction, mediastinitis, peritonitis, or even death [36].

FBs with a sharp edge are associated with an increased risk of impaction and perforation, followed by extraluminal migration, abscess, peritonitis, liver, bladder, heart, or lung penetration, carotid artery lesion, aorto-esophageal fistula, and death.

Coins can cause significant morbidity and mortality when retained in the GI tract for long periods, such as unilateral vocal cord paralysis, esophageal impaction, tracheoesophageal fistulas, esophageal perforation, esophageal ulceration, peri-esophagitis, esophageal or tracheal stenosis, mediastinitis, pneumothorax, and pyothorax [37].

## 8. Conclusions

Although FB ingestion in children typically does not result in complications, it can sometimes lead to serious, even fatal outcomes.

Therefore, it is important to consider the diagnosis in cases of nonspecific symptoms, even in the absence of a relevant medical history, and to proceed with appropriate evaluation and prompt treatment to prevent serious consequences. It is also important to know which patients require hospitalization and monitoring, but especially to identify situations that require immediate medical intervention accurately.

Patients with preexisting gastrointestinal disorders or those who present with airway compromise, hemodynamic instability, hypersalivation, recurrent vomiting, GI bleeding, or other signs of GI obstruction and perforation are considered emergencies.

However, certain high-risk objects require urgent endoscopic removal within the first two hours, even in the absence of symptoms, to prevent serious complications such as perforation, obstruction, or fistula formation: button batteries or sharp objects lodged in the esophagus, sharp objects or multiple magnets in the stomach or duodenum, coingestion of magnets and button batteries or metal components, and expandable FBs.

Prevention plays a crucial role; therefore, it is important to raise awareness among parents and caregivers that dangerous objects must be kept out of the reach of children.

## Figures and Tables

**Table 1:** Recommended timing for FB extraction [20,21,22].

Urgency Degree	GI Tract Location	Ingested Object
**Emergency** (**requires intervention in <4 h**)	Esophagus	Button batteries Sharp objects Any FB causing complete esophageal obstruction (inability to manage secretions) Any FB causing airway compromise (stridor, choking, severe respiratory distress)
	Stomach	Symptomatic button batteries Symptomatic multiple magnets Sharp objects
	Duodenum	Sharp objects Symptomatic multiple magnets Blunt FB causing symptoms
**Urgency** (**requires intervention in <24 h**)	Esophagus	Non-sharp/non-battery objects lodged in the esophagus without complete obstruction Superabsorbent objects Asymptomatic or mildly symptomatic blunt FB
	Stomach	Button batteries ≥ 20 mm in size in patients aged <5 years Multiple magnets Coingestion of batteries and magnets Objects wider than 2.5 cm or longer than 6 cm in symptomatic patients Blunt FB causing symptoms Superabsorbent objects with or without symptoms
	Duodenum	Multiple magnets Coingestion of batteries and magnets Objects wider than 2.5 cm or longer than 6 cm in symptomatic patients
**Early elective** (**requires intervention in <48 h**)	Stomach	Objects wider than 2.5 cm or longer than 6 cm in asymptomatic patients Button batteries ≥ 20 mm in size in patients aged >5 years Asymptomatic button batteries passed into the stomach can be monitored for 48 h
**Elective** (**requires intervention in >48 h**)	Duodenum	Cylindrical batteries can be monitored for 7–14 days Blunt FB failing to pass spontaneously after 4 weeks

## Data Availability

No new data were created or analyzed in this study. Data sharing is not applicable to this article.

## Abbreviations

The following abbreviations are used in this manuscript:

AIGO	Italian Association of Hospital Gastroenterologists and Endoscopists
CT	Computed tomography
ENT	Ear, nose, and throat
ESGE	European Society of Gastrointestinal Endoscopy
FB	Foreign body
GI	Gastrointestinal
MRI	Magnetic Resonance Imaging
NASPGHAN	North American Society for Pediatric Gastroenterology, Hepatology and Nutrition
NPO rules	Nil per os/nothing by mouth rules (standard pre-anesthesia fasting rules)
SIGENP	Italian Society of Pediatric Gastroenterology, Hepatology, and Nutrition
X-ray	Radiographic investigation
